# P02.43. A Mediterranean-style, low-glycemic diet plus phytonutrient rich medical food improves cardiovascular risk variables in women with metabolic syndrome

**DOI:** 10.1186/1472-6882-12-S1-P99

**Published:** 2012-06-12

**Authors:** R Lerman, M McIntosh, M Fernandez, W Najm

**Affiliations:** 1 Metagenics Inc., Gig Harbor, USA; 2 Department of Emergency Medicine, University of Florida, Jacksonville, Jacksonville, USA; 3 Department of Nutritional Sciences, University of Connecticut, Storrs, USA; 4 Department of Medicine, University of California, Irvine, Irvine, USA

## Purpose

Metabolic syndrome (MetS) is a growing public health concern and effective dietary intervention programs could make a tremendous impact on slowing disease progression. To assess the benefits of a dietary program on cardiometabolic diease risk variables, a 12-week, randomized controlled trial of overweight and obese women with MetS was conducted.

## Methods

Participants consumed a Mediterranean-style, low-glycemic-load diet (control arm, n = 44), or the same diet plus a medical food (UltraMeal PLUS 360, Metagenics Inc.) containing phytosterols, soy protein, and extracts from hops and acacia (intervention arm, n = 45). Fasting blood samples were analyzed at baseline, week 8, and week 12 for plasma lipids, apolipoproteins, and homocysteine. Dietary records were collected and analyzed.

## Results

Reduction in fat and sugar intake (p <.001 for both) was observed and increases in docosahexaenoic acid and eicosapentaenoic acid intake (p <.001 for both) were recorded, consistent with the prescribed diet. Regarding MetS variables, decreases in waist circumference, systolic and diastolic blood pressure, and plasma triglycerides in all subjects (p <.001 for all) were observed, with no differences between arms. Plasma low-density lipoprotein cholesterol, non-high-density lipoprotein cholesterol, apolipoprotein (apo) B, and apo B/apo A1 were reduced over the 12-wk study, but to a greater extent in the intervention arm (p <.05 for all), indicating the medical food had an effect in altering lipoprotein metabolism. Further, medical food intake was associated with reduced plasma homocysteine (p <.01), compared to the control arm.

## Conclusion

A Mediterranean-style, low-glycemic-load diet effectively reduced cardiovascular risk factors associated with MetS. Addition of medical food resulted in an improved lipoprotein profile and lowered plasma homocysteine.

